# Supplementary figures and images for: Embedding covariate adjustments in tree-based automated machine learning for biomedical big data analyses
Source: BMC Bioinformatics. 2020 Oct 1;21:430. doi: 10.1186/s12859-020-03755-4 (PMC7528347; doi:10.1186/s12859-020-03755-4)

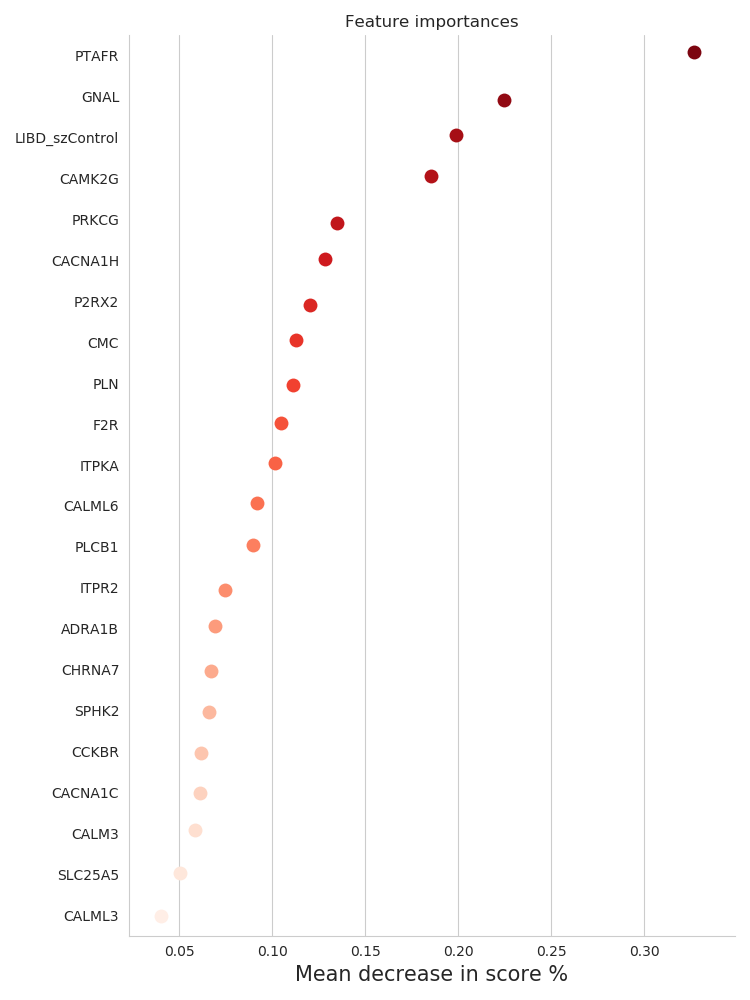

Supplement: Supplementary file 1 — Additional file 1. Permutation importance from 100 runs of resAdj TPOT on the PsychENCODE data set. The top 20 genes (features) and 2 covariates are shown. The gene names are displayed on the y-axis and the weighted (by testing score) averages of the mean score decrease as a percentage of the score are displayed on the x-axis. [file 12859_2020_3755_MOESM1_ESM.png]

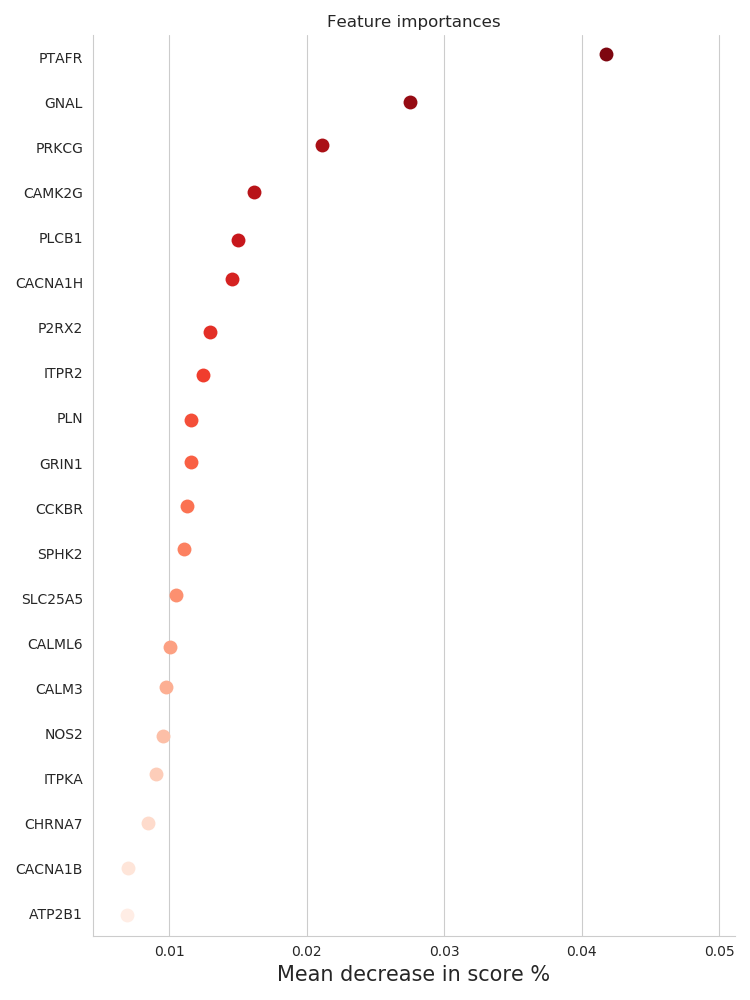

Supplement: Supplementary file 2 — Additional file 2. Permutation importance from 100 runs of classic TPOT on the PsychENCODE data set. The top 20 genes (features) are shown. The gene names are displayed on the y-axis and the weighted (by testing score) averages of the mean score decrease as a percentage of the score are displayed on the x-axis. [file 12859_2020_3755_MOESM2_ESM.png]
