# Supplementary material for: Embedding covariate adjustments in tree-based automated machine learning for biomedical big data analyses
Source: BMC Bioinformatics. 2020 Oct 1;21:430. doi: 10.1186/s12859-020-03755-4 (PMC7528347; doi:10.1186/s12859-020-03755-4)
Supplement: Supplementary file 4 — Additional file 4. Additional methods. Details for the leakage-free covariate adjustment. [file 12859_2020_3755_MOESM4_ESM.pdf]

Additional file 4

Leakage-free covariate adjustment

Let v be the target variable or feature which we want to adjust by covariates C_1, C_2, ..., C_r. Denote by v_i and C_{i1}, C_{i2}, ..., C_{ir} the observed values of v and the Cs for subject i.

Suppose we are given a cross-validation split of the subjects into a training and a testing set.

Continuous v

If v is continuous, we use the **training set** to fit a linear regression model:

$$v = \beta_0 + \beta_1 C_1 + \beta_2 C_2 + \ldots + \beta_r C_r.$$

For each subject i **(whether in the training or testing set)**, we subtract the model-fitted values from v_i:

$$v_i^{\text{adj}} = v_i - (\hat{\beta}_0 + \hat{\beta}_1 C_{i1} + \hat{\beta}_2 C_{i2} + \ldots + \hat{\beta}_r C_{ir}).$$

Binary v

If v is binary with values are 0 and 1, we use the **training set** to fit a logistic regression model:

$$\text{logit}(\pi) = \beta_0 + \beta_1 C_1 + \beta_2 C_2 + \ldots + \beta_r C_r$$

where $\pi = Prob(v = 1)$.

For each subject i **(whether in the training or testing set)**, we subtract the model-fitted values from the observed outcomes:

$$v_i^{\text{adj}} = v_i - \hat{\pi}_i$$

where

$$\hat{\pi}_i = \frac{\exp(\hat{\beta}_0 + \hat{\beta}_1 C_{i1} + \hat{\beta}_2 C_{i2} + \ldots + \hat{\beta}_r C_{ir}}{1 + \exp(\hat{\beta}_0 + \hat{\beta}_1 C_{i1} + \hat{\beta}_2 C_{i2} + \ldots + \hat{\beta}_r C_{ir})}.$$

Multiclass ordinal v

This is a simple extension of the binary case. If v is multiclass ordinal with values $0, 1, \ldots, K$, we use the **training set** to fit a multinomial logistic regression model, deriving values $\hat{\pi}_k$ for $k = 1, \ldots, K$.

For each subject i **(whether in the training or testing set)**, we subtract the model-fitted values from the observed outcomes:

$$v_i^{\text{adj}} = v_i - \sum_{k=1}^{K} \hat{\pi}_{ki} k.$$
