# Supplementary material for: Embedding covariate adjustments in tree-based automated machine learning for biomedical big data analyses
Source: BMC Bioinformatics. 2020 Oct 1;21:430. doi: 10.1186/s12859-020-03755-4 (PMC7528347; doi:10.1186/s12859-020-03755-4)
Supplement: Supplementary file 5 — Additional file 5. resAdj TPOT pre-processor output. Structure and column description of the output from this pre-processor. [file 12859_2020_3755_MOESM5_ESM.pptx]

## Slide 1
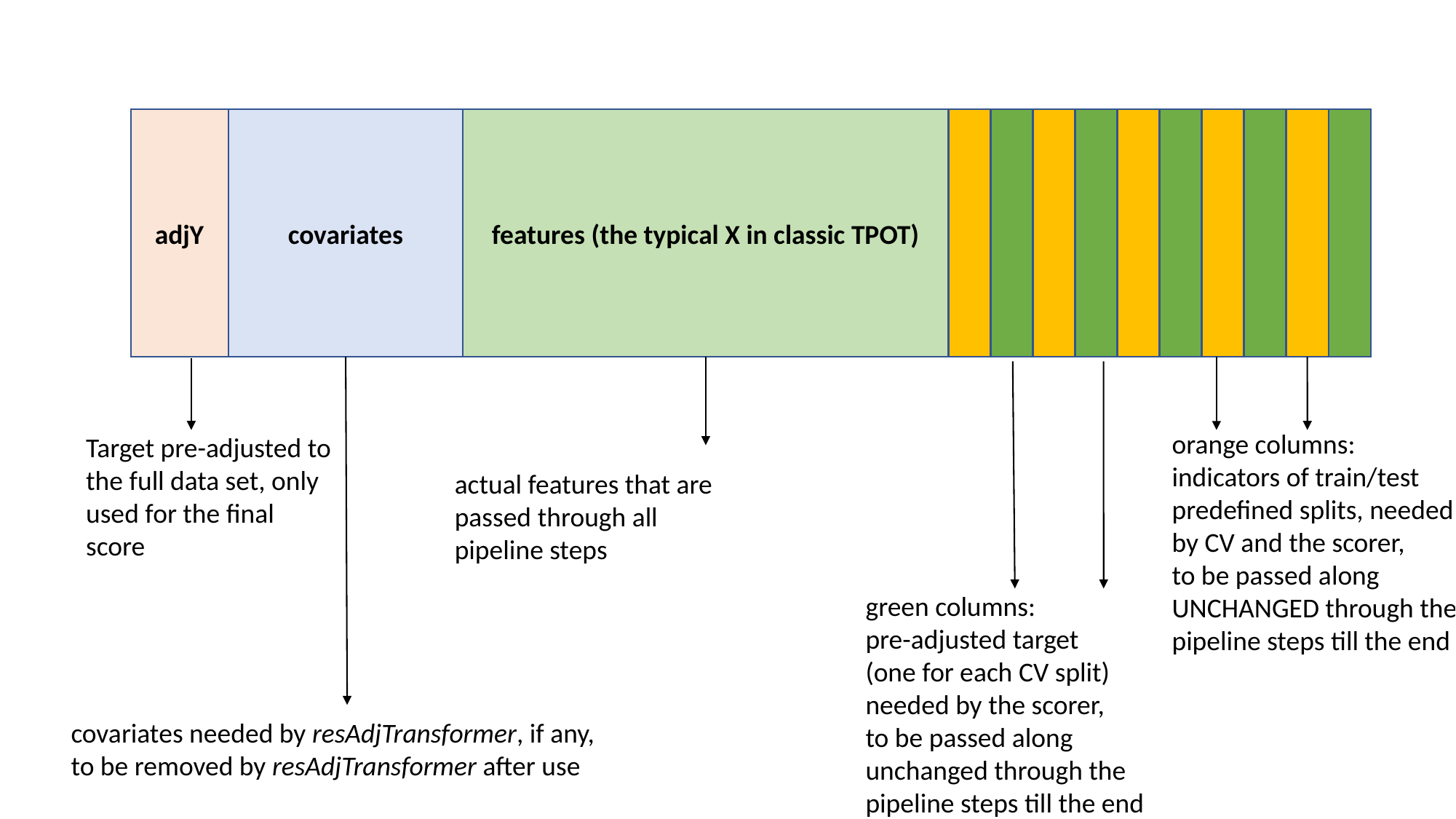

adjY
covariates
features (the typical X in classic TPOT)
orange columns:
indicators of train/test
predefined splits, needed
by CV and the scorer,
to be passed along
UNCHANGED through the
pipeline steps till the end
Target pre-adjusted to
the full data set, only
used for the final
score
actual features that are
passed through all
pipeline steps
green columns:
pre-adjusted target
(one for each CV split)
needed by the scorer,
to be passed along
unchanged through the
pipeline steps till the end
covariates needed by resAdjTransformer, if any,
to be removed by resAdjTransformer after use
